# Oil-Based Mud Waste as a Filler Material in LDPE Composites: Evaluation of Mechanical Properties

**DOI:** 10.3390/polym14071455

**Published:** 2022-04-02

**Authors:** Shohel Siddique, Adam Novak, Emin Guliyev, Kyari Yates, Pak Sing Leung, James Njuguna

**Affiliations:** 1 Advanced Materials Research Group, School of Engineering, Robert Gordon University, Riverside East, Garthdee Road, Aberdeen AB10 7GJ, UK; s.a.siddique@rgu.ac.uk (S.S.); a.a.novak@rgu.ac.uk (A.N.); e.guliyev@rgu.ac.uk (E.G.); 2 School of Pharmacy and Life Sciences, Robert Gordon University, Aberdeen AB10 7GJ, UK; k.yates@rgu.ac.uk; 3 Department of Mechanical and Construction Engineering, Northumbria University, Ellison Place, Newcastle upon Tyne NE1 8ST, UK; ps.leung@northumbria.ac.uk; 4 National Subsea Centre, 3 International Ave, Dyce, Aberdeen AB21 0BH, UK

**Keywords:** polymer–clay nanocomposites, filler dispersion, filler loading, interfacial adhesion, mechanical properties

## Abstract

Traditionally, the drilling waste generated in oil and gas exploration operations, including spent drilling fluid, is disposed of or treated by several methods, including burial pits, landfill sites and various thermal treatments. This study investigates drilling waste valorisation and its use as filler in polymer composites. The effect of the poor particle/polymer interfacial adhesion bonding of the suspended clay in oil-based mud (OBM) slurry and the LDPE matrix is believed to be the main reason behind the poor thermo-mechanical and mechanical properties of low-density polyethylene (LDPE)/OBM slurry nanocomposites. The thermo-mechanical and mechanical performances of LDPE)/OBM slurry nanocomposites without the clay surface treatment and without using compatibilizer are evaluated and discussed. In our previous studies, it has been observed that adding thermally treated reclaimed clay from OBM waste in powder form improves both the thermal and mechanical properties of LDPE nanocomposites. However, incorporating OBM clay in slurry form in the LDPE matrix can decrease the thermal stability remarkably, which was reported recently, and thereby has increased the interest to identify the mechanical response of the composite material after adding this filler. The results show the severe deterioration of the tensile and flexural properties of the LDPE/OBM slurry composites compared to those properties of the LDPE/MMT nanocomposites in this study. It is hypothesised, based on the observation of the different test results in this study, that this deterioration in the mechanical properties of the materials was associated with the poor Van der Waals force between the polymer molecules/clay platelets and the applied force. The decohesion between the matrix and OBM slurry nanoparticles under stress conditions generated stress concentration through the void area between the matrix and nanoparticles, resulting in sample failure. Interfacial adhesion bonding appears to be a key factor influencing the mechanical properties of the manufactured nanocomposite materials.

## 1. Introduction

The improvement of specific features, such as the mechanical, thermal, gas barrier, heat resistance and flame retardancy, in polymer matrices has led to an increasing interest in polymer nanocomposites research [1,2,3,4,5,6]. Adding layered silicates even at very low concentrations (<5 wt%) in polymer matrices, predominantly in polar polymers, improved the material properties significantly [7,8,9,10,11]. An effective polymer–clay affinity leads to strong polymer–clay interfacial adhesion, which helps to disperse the clays by delaminating the platelets at the nanoscale level [12,13,14]. Although interfacial adhesion between polar polymers and clay platelets is successful to some extent, the interfacial adhesion between non-polar polymers, such as polyethylene and clay platelets, is still very poor and limited [15,16,17]. Different researchers have applied various techniques, including utilising compatibilizers and coupling agents in the nanocomposites manufacturing process [18,19,20,21]. Considering the poor performances/results obtained from dispersing clay platelets into non-polar polymer matrices, an effective and suitable process for manufacturing cheap and eco-friendly nanocomposites is still an attractive area of research [22,23]. In addition, the source of clay minerals is limited: the reclamation of clay mineral from different sources of industrial operations, such as the oil and gas industry, is a niche area of recent research. 

In the oil and gas exploration industry, bentonite clay is typically used in oil-based drilling fluid as an additive to correct the viscosity. The oil-based mud is a complex mixture of different chemicals, including weighting agents, alkaline chemicals, lignosulfonates, polymers/viscosifiers, emulsifiers/detergent, base oils, etc. [22,23]. A series of “physical and chemical” treatments are applied to modify the surface of clay, aiming to disperse the clay platelets in the continuous (organic) phase. Cationic surfactants are used to modify the clay surface by replacing positive cations such as “Na, K, Al etc with higher amounts of ammonium cations”, which are adsorbed on the clay surface, suggesting an increasing affinity between the clay surface and base oil [23,24]. 

All the conventional drilling waste treatment methods, including disposal in burial pits and landfill sites, stabilisation and solidification, are associated with cost, time, space requirements and are potentially environmentally hazardous [25]. The existing drilling waste management techniques in the oil and gas industry are facing a major challenge as these techniques hinder the economic robustness and are also very limited in terms of protecting the environment. To meet strict environmental regulations, a sustainable and effective waste management strategy is a major demand now in the oil and gas industry. Fortunately, advancements in waste treatment operations demonstrate improved clean-up operations in the oil and gas industry. Although these processes are successful to some extent in terms of meeting the discharge/disposal regulations, in the long-run, these techniques may pass the pollutants from one stage to another stage or to a secondary level of environmental pollution. The potential solution to this global problem is either to destroy these hazardous chemicals completely, which is a major challenge, or to make use of them for beneficial purposes. This recycling theme promotes a new window to turn the accumulated hazardous wastes in the oil and gas industry into value-added products. 

The aim of this research is to utilise and convert the oil-based mud (OBM) waste into useful fillers for applications in engineering materials. To utilise this waste material, which is considered a sink of pollutants, it is very important to understand the sources of drilling fluid wastes, chemical composition and characterisation of these wastes to design an effective treatment process that helps to develop a safe polymer nanocomposite manufacturing process. Investigating the literature on the chemical composition of OBM wastes, bentonite clay comprises a significant percentage of drilling fluid waste [7,16]. In addition, the initial characterisation of OBM waste confirms the presence of different clay minerals that have significant importance in developing new composite material. Clay/nanoclay is an important component in structural materials by improving the mechanical and thermal properties of polymer/clay nanocomposite materials, which is comprehensively reported in the literature. Although clay/nanoclay improves the mechanical and thermal properties of polymer/clay nanocomposite materials, there is no information available in the literature regarding how the recovered nanoclay from OBM waste influences the mechanical and thermal properties of polymer nanocomposite materials. The motivation of this study is to identify the influential factors affecting the mechanical and thermal properties of LDPE and PA6 matrix materials manufactured by utilising nanoclay fillers recovered from OBM waste.

Consequently, we investigated the potential of utilising reclaimed layered silicate from oil-based mud (OBM) waste to influence the properties of engineering nanocomposite materials. In our previous works, in-depth structural, morphological, thermo-mechanical and thermal investigations were carried out on nanocomposite materials manufactured from heat-treated OBM waste-reinforced polymers [7,16]. Different types of clay minerals, such as halloysite, muscovite, anorthite, montmorillonite, kaolinite, etc., remained strongly suspended in the OBM waste slurry [7]. It is anticipated that the cations existing in the clay mineral layers would be replaced, intercalated or compensated by exchangeable cations by amines or quaternary ammonium salts, which are believed to be used in the OBM formulation. In our previous studies [16,26], using the same polymer and OBM clay in both slurry and dry powder form confirms the even distribution of the fillers in the polymer matrix. This observation inspired us to utilise the organophilic nature of clay surfaces that exist in OBM waste in its natural condition and to investigate the effect of this favourable distribution nature of filler in influencing different mechanical properties of LDPE/OBM slurry nanocomposites. Further, the conventional oil-based mud treatment processes are expensive, time-consuming and, additionally, cause secondary pollution. A sustainable approach to utilise the applicable minerals that exist in this waste is our goal. Thermally treated oil-based mud fillers were successfully embedded into LDPE and PA6 polymer matrices, which was reported in our previous publications [16,26]. It is essential in waste processing, especially hazardous waste management processes, to use materials with the lowest possible degree of processing. This principle motivated us to investigate the effect of this raw OBM slurry waste influencing the thermo-mechanical, structural, morphological and mechanical properties of LDPE nanocomposites. 

The present work is an extension of our previous studies and focuses on investigating the effect of this novel filler (from the oil-based mud) as a slurry without any pre-treatment on the thermo-mechanical and mechanical properties of the LDPE/OBM slurry nanocomposites.

## 2. Materials and Methods

### 2.1. Materials and Samples Preparation

Lupolen 1800S manufactured by Lyondellbasell industries Ltd., Milton Keynes, UK with the trade name of LDPE was supplied by Northern Polymers and Plastics Ltd., Crewe, UK. The molecular weight and melting temperature of this LDPE are 356 kg/mol (polydispersity 6.7) and 106 °C, respectively, and it has a melt flow index of 20 g/10 min and density of 917 kg/m^3^. Montmorillonite (MMT, chemical formula: H_2_Al_2_(SiO_3_)_4_·nH_2_O with surface area 220–270 m^2^/g) K10 as a reference filler was supplied by Sigma-Aldrich, Gillingham, UK. The spent OBM waste slurry was obtained from a local oil and gas service company in Aberdeen, UK. To characterise the solid content in this OBM slurry, the petroleum hydrocarbon was eliminated by using thermal treatment process. To obtain the solid residue, the OBM slurry is heated sporadically via the following stages: 50 °C for 12 h (1st heating) followed by 80 °C for a further 12 h (2nd heating); finally, the residue is heated at 700 °C for 12 h (3rd heating). For subsequent analysis, this solid residue was crushed into smaller pieces using a grinder, followed by a further size reduction to produce powder by using an IKA UltraTurrax ball mill.

### 2.2. LDPE/OBM Slurry Nanocomposite Manufacturing Process

**Figure 1:**
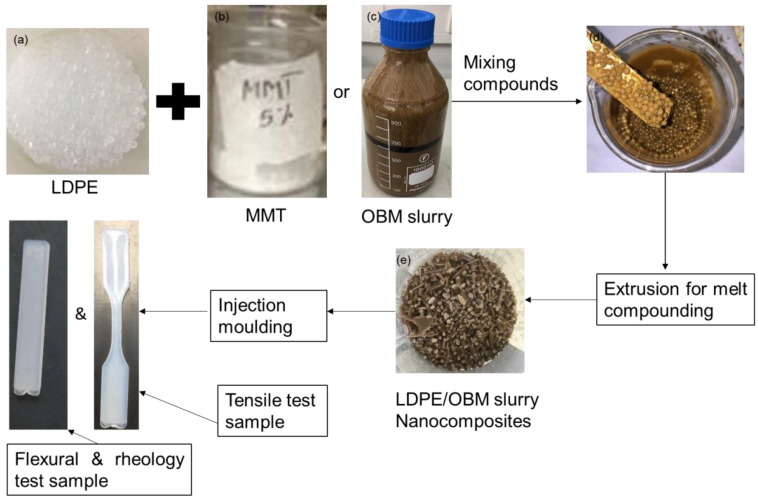
Flow diagram of LDPE/MMT and LDPE/OBM slurry nanocomposites sample manufacturing process.

LDPE/MMT and LDPE/OBM slurry nanocomposites were manufactured by following a step-by-step process that is schematically presented in Figure 1. Briefly, LDPE polymer, OBM waste slurry and MMT were kept in a convection oven at 90 °C for 12 h to remove any moisture prior to melt compounding. LDPE was mixed with OBM waste slurry and the MMT filler at different weight percentages of 0, 2.5, 5, 7.5 and 10 wt%. This mixing process of filler with LDPE was performed in a fume hood and left for at least 30 min to reduce any volatile hydrocarbons in the OBM slurry. 

A twin screw co-rotating extruder (TwinTech extrusion Ltd., Staffordshire, UK, was used to manufacture LDPE/MMT and LDPE/OBM slurry nanocomposites. The temperatures were different in five heating zones, such as 1st zone (120 °C), 2nd zone (200 °C), 3rd zone (210 °C), 4th zone (200 °C) and die/5th zone (200 °C), and the screw diameter was 25 mm. The compounds were manufactured at the speed of 60 rpm. The manufactured compound strands were passed through the cold-water bath followed by sizing in a pellet form by using a pelletiser. These compound pellets were stored in thermally insulated bags to protect them from moisture attack. Different nanocomposite samples were made from these compound pellets using an injection moulding machine, adjusting the barrel temperature to 230 °C with a moulding pressure of 100 bar. The test samples were stored in thermally insulated bags before performing the mechanical tests and dynamic mechanical analysis (DMA). Nanocomposite test samples were dried at 45 °C for 12 h in a convection oven before further tests and analyses of the samples were made.

### 2.3. Sample Testing and Characterisation

#### 2.3.1. Particle Size and Morphology Analysis

Colloidal particles under Brownian motion generate a discontinuous scattered intensity due to laser irradiation, which reflects the size and shape of the particles. Following this principle, the *Z*-average diameters of MMT and OBM clay were determined on a Malvern Zetasizer (DTS Ver. 5.10). The morphologies of MMT and OBM slurry clay platelets were analysed along with MMT and OBM slurry embedded into the LDPE matrix by using a Zeiss EVO LS10 Scanning Electron Microscope (SEM, Carl Zeiss Microscopy Deutschland GmbH, Oberkochen, Germany). The samples were fractured following a cryogenic sampling technique and were gold–palladium-coated using sputter deposition for 2 min prior to fitting in the SEM for observation. 

#### 2.3.2. Chemical Structure and Thermal Analysis

Attenuated total reflectance-Fourier transform infra-red (ATR-FTIR) spectroscopy analysis was performed to identify and evaluate the changes in chemical structure of manufactured LDPE/MMT and LDPE/OBM slurry nanocomposites relative to that of unfilled LDPE matrix. A Thermo Scientific Nicolet iS10 ATR-FTIR Spectrometer was used to investigate the chemical structure by performing 32 scans between 4000–400 cm^−1^ following background measurement. Thermo Scientific™ OMNIC™ Specta Software was used to gather clear visual data and as an identification and interpretation tool for material characterisation as well as comparison to a dedicated spectral library database. 

A TA Q100 instrument under a nitrogen environment was used for performing differential scanning calorimetry (DSC) thermal analysis by following a heat–cool–heat procedure with the temperature ramp of 10 °C/min from −20 °C to 250 °C. A TA Q500 instrument was used to perform thermogravimetric analysis (TGA) to identify the degradation and decomposition nature of the materials. The temperature was set on ramp mode from room temperature (15 °C) to 1000 °C at a rate of 10 °C/min.

#### 2.3.3. Mechanical Testing

Tensile tests were carried out by following ASTM method D-638 [27]. Five specimens for each type of nanocomposite were tested at room temperature (20 ± 1 °C) and 71% humid environment. An Instron 3382 universal testing machine with Bluehill 3 software was used to perform the tensile test at constant strain rate of 2 mm/min. The manufactured LDPE with different wt% of MMT and OBM slurry fillers was prepared as dog-bone shaped samples. Flexural experiments were carried out according to ISO 14125 test standard. All tests were performed at a constant strain rate of 2 mm/min using the same equipment as for tensile test on the manufactured nanocomposite materials reinforced with different wt% of MMT and OBM slurry fillers. A set of five samples were tested, and the average data are considered to identify the flexural properties of each material.

Thermo-mechanical characterisation was performed by following the same method described in our previous study [16]. Briefly, an oscillatory shear rheometer (AR 1000, TA Instruments) was used to identify the viscoelastic properties as a function of temperature from 0 to 90 °C, with a heating rate of 3 °C/min. Parallel plate geometry of 8 mm diameter was adjusted to 3.1 mm gap to hold the sample between plate and geometry. The analysis was performed at a fixed frequency mode of 1 Hz, and a strain rate of 0.2%.

A multi-sample statistical analysis of measured data was conducted, and the 95% confidence interval of the mean value using the procedure given in ISO 2602 was employed for the test results analysis.

## 3. Results and Discussion

### 3.1. Particle Size, Morphology Study and Chemical Structure Analysis

**Figure 2:**
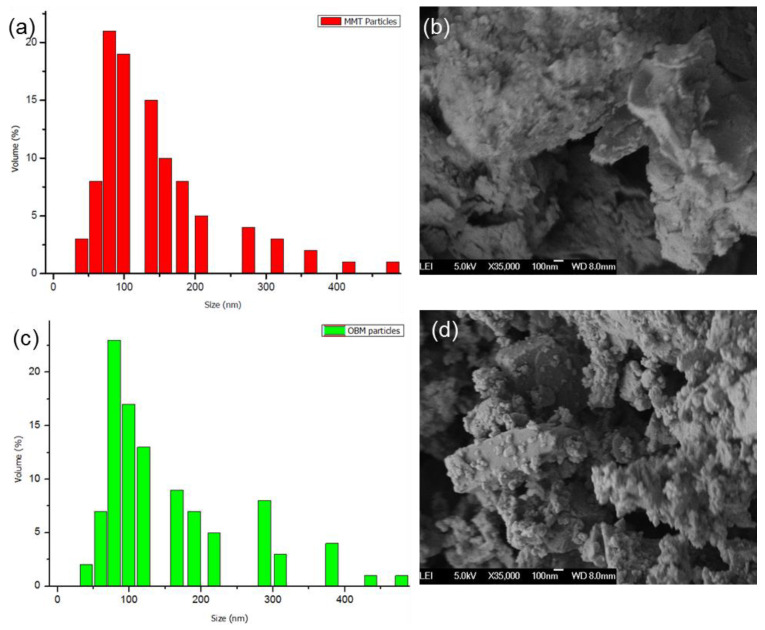
Particle size distribution (**a**,**c**) and SEM images (**b**,**d**) of the representative MMT and OBM clays.

The average particle diameters of the MMT and OBM clay were identified to be in the range of 139.61 and 151.51 nm, respectively. These results represent the fine particle size of both the MMT and OBM clay. The particle size distribution graphs in Figure 2a,c showed a very similar pattern. However, whereas the particle distribution graph of the MMT showed only one narrow peak, the particle distribution graph of the OBM clay showed two more additional peaks at higher size diameter ranges. The specific surface area (SSA) of both the MMT and OBM clay were also identified in this study, which were 341 ± 35 m^2^g^−1^ and 675 ± 41 m^2^g^−1^, respectively.

The SEM micrographs in Figure 2b,d showed that the platelets were both in the nano-size range. However, the MMT clay platelets appeared to be loosely bound to each other, with smooth surfaces. In the case of the OBM clay, the clay platelets were closely stacked together and showed rough surfaces with dissimilar shapes. The dissimilarity in the shapes can be explained in this case as a result of the presence of different clay minerals, including metals. 

In addition to the morphological analysis of the MMT and OBM clay, the SEM micrographs were used to analyse and compare the changes in the morphological structure of the MMT and OBM slurry embedded into the LDPE matrix.

**Figure 3:**
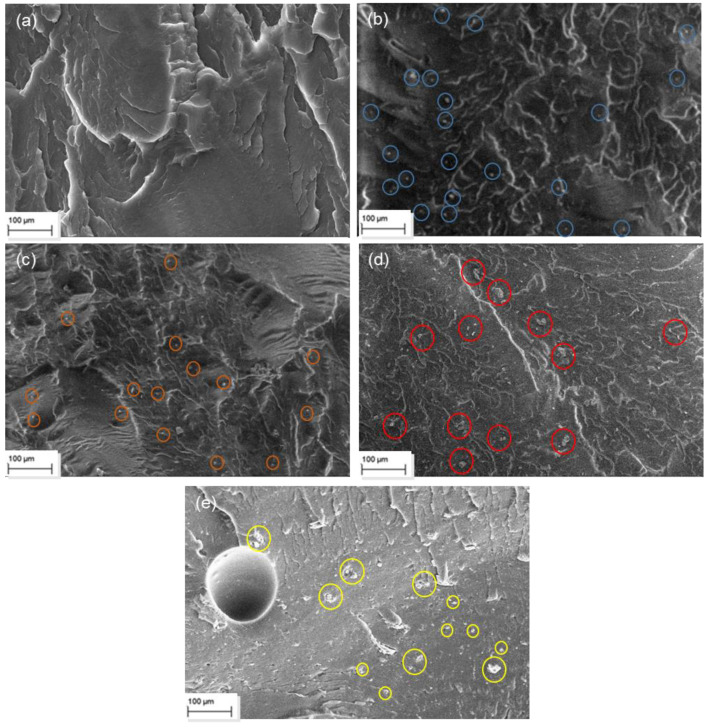
SEM images of (**a**) neat LDPE; (**b**) LDPE with 2.5 wt% MMT; (**c**) LDPE with 5 wt% MMT; (**d**) LDPE with 7.5 wt% MMT and (**e**) LDPE with 10 wt% MMT.

**Figure 4:**
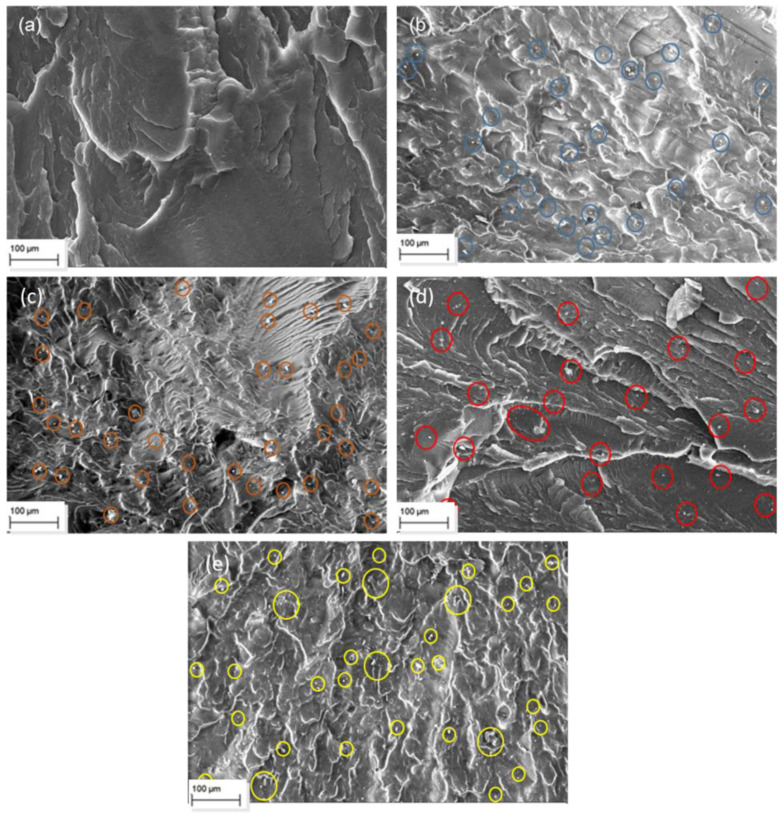
SEM images of (**a**) neat LDPE; (**b**) LDPE with 2.5 wt% OBM slurry; (**c**) LDPE with 5 wt% OBM slurry; (**d**) LDPE with 7.5 wt% OBM slurry and (**e**) LDPE with 10 wt% OBM slurry.

Figure 3 and Figure 4 showed the cavities and uneven surfaces, which are due to the cryogenic fractures of the samples. A higher degree of filler dispersion is observed in the LDPE/OBM slurry nanocomposite samples (Figure 4) compared to the MMT dispersion in the LDPE/MMT nanocomposites (Figure 3), predominantly in the higher (7.5 and 10 wt% loading in this study) filler content samples in Figure 3e and Figure 4e. Figure 3d,e shows the tendency of MMT agglomeration in the LDPE matrix, whereas Figure 4d,e shows the uniform distribution of the OBM slurry in the LDPE matrix. Both Figure 3 and Figure 4 show the chain formation around the particulate materials due to the presence of LDPE chains, which was also observed regarding similar patterns of polyamide chains on the palygorskite by Saleh et al. (2018) [28]. Furthermore, the energy dispersive spectroscopy analysis of these samples from our previous study [26] also supports the above-mentioned dispersion and distribution behaviour of the OBM slurry in the LDPE matrix.

**Figure 5:**
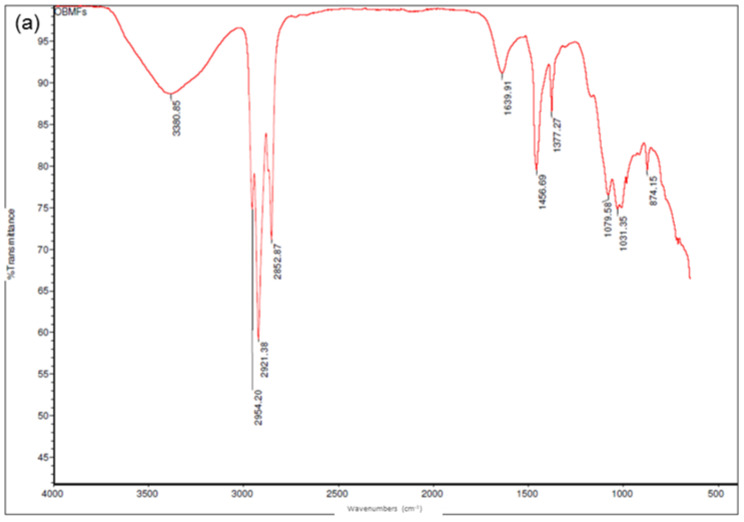

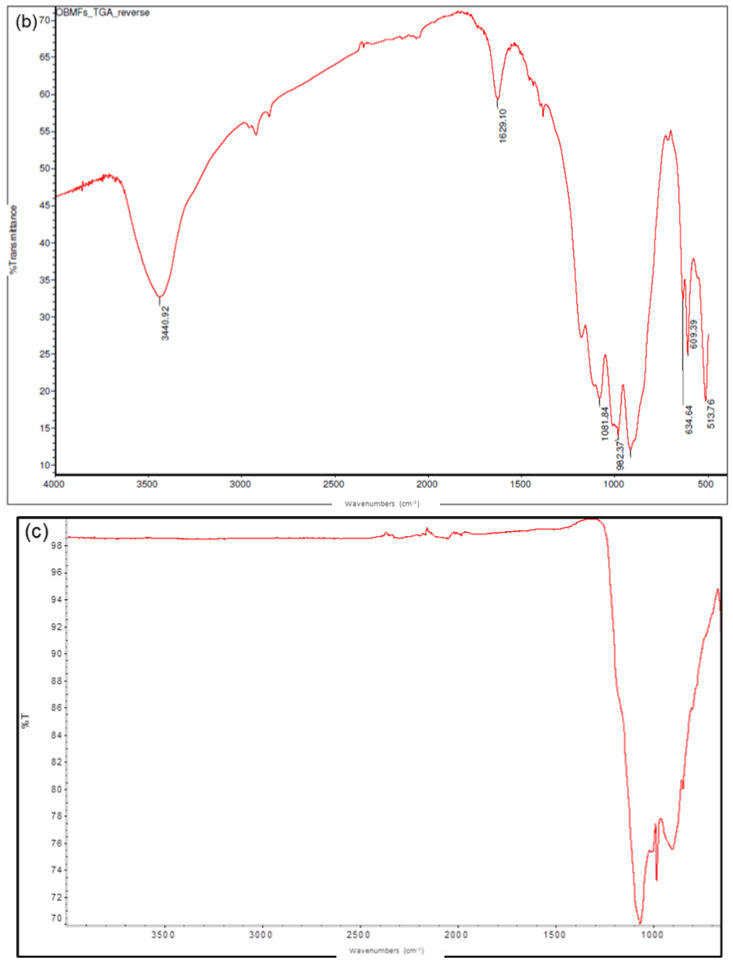
ATR-FTIR full scale spectra of (**a**) OBM waste slurry; (**b**) OBM waste residue after TGA analysis and (**c**) OBM waste dry powder.

Figure 5a shows the presence of alkyl C-H bonds (~2800–3000 cm^−1^) in the OBM slurry, which corresponds to the hydrocarbon presence in the substance. Figure 5b,c illustrates different peaks corresponding to different chemical bonds in the OBM waste after the TGA and in the OBM waste powder. A broad band near 3380 cm^−1^ corresponds to the H-O-H vibrations of the adsorbed water. The clay minerals in the OBM waste show Si-O stretching and bending as well as OH bending absorptions in the range of 1300 to 400 cm^−1^. Variations in the layers’ arrangements are reflected in the shapes and positions of the bands. Strong band peaks at 1120 to 1000 cm^−1^ correspond to the Si-O stretching vibrations of kaolinite and dickite. The main Si-O band peak corresponding to chrysotile is observed at a lower frequency of 980 cm^−1^. The peak at 1080 cm^−1^ is indicative of montmorillonite, whereas the peak at 980 cm^−1^ corresponds to the saponite minerals in the OBM waste. The band peaks in the region of 980 to 900 cm^−1^ represent the dioctahedral minerals, while the band in the 700 to 600 cm^−1^ range corresponds to the trioctahedral minerals. The band peak at 600 cm^−1^ represents the presence of Mg_3_(OH)_2_. The peak at 916 cm^−1^ reflects the partial substitution of octahedral Al by Mg in the montmorillonite. 

The resulting spectra from the ATR-FTIR analysis of the OBM waste slurry and its solid contents are presented in Figure 5.

### 3.2. Chemical Structure Analysis of LDPE/MMT and LDPE/OBM Slurry Nanocomposites

**Figure 6:**
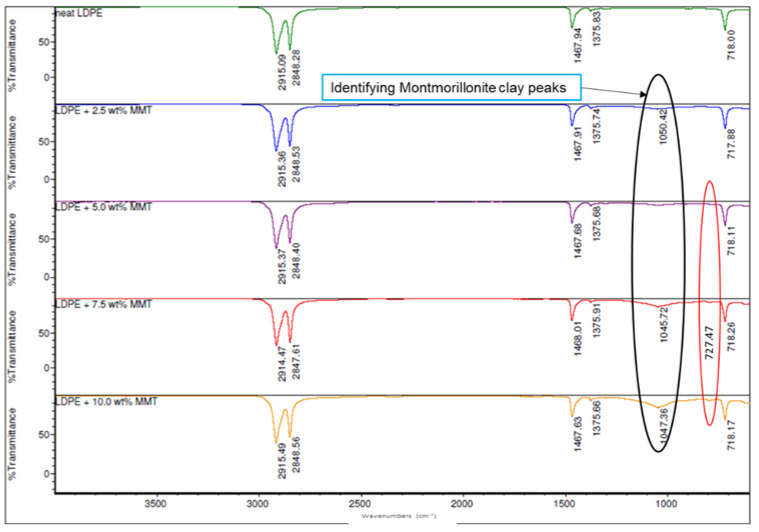
Comparison of ATR-FTIR spectra of LDPE and LDPE/MMT nanocomposites.

**Figure 7:**
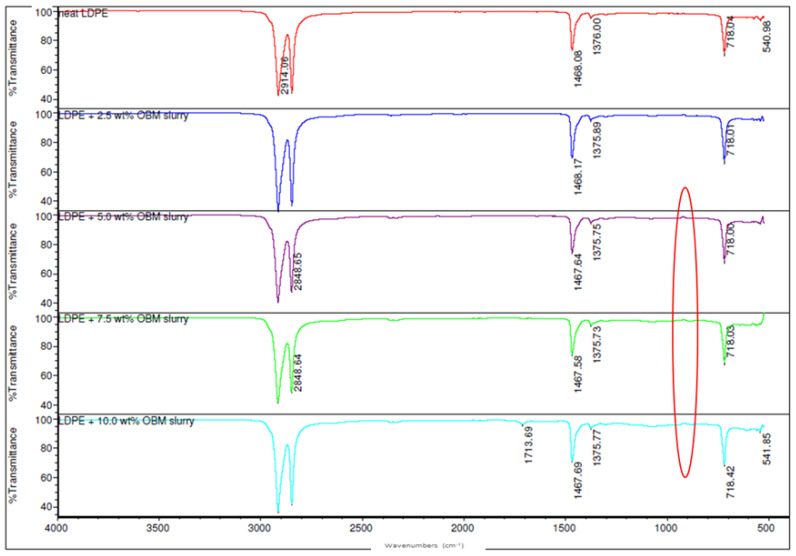
Comparison of ATR-FTIR spectra of LDPE and LDPE/OBM slurry nanocomposites.

The LDPE polymer, LDPE/MMT and LDPE/OBM slurry nanocomposites’ spectra were collected for the filler range of 0 to 10 wt% to determine if the presence of the clay platelets could be established. The FTIR analysis identifies the functional groups by the accumulated vibrational modes. The detailed band assignments of the LDPE and LDPE/OBMFs nanocomposites were reported in our previous article [16]. Similar peaks at 2914 and 2848 cm^−1^ to those found with the thermally treated OBM waste slurry used in Ref. [16], corresponding to the CH_2_ asymmetric stretching vibration [29], and at 1468 and 1375 cm^−1^, highlighting the presence of the bending and rocking (asymmetric and symmetric vibration, respectively) vibration of CH_2_ and the presence of carbonate ions, respectively, were observed (Figure 6 and Figure 7) [30]. However, a small shift to a lower wavenumber is noticeable for the MMT clay minerals, which is shown in Figure 6. It is evident that the degree of IR absorption due to new chemical bonding increases with the incremental loading of the MMT fillers in the nanocomposites. 

Surprisingly, there were no new peaks formed due to the addition of the OBM slurry as a filler in the LDPE polymer except a small peak at 1045 cm^−1^ (Figure 7), likely corresponding to the silica–aluminium and aluminosilicates [31] in the LDPE with 10 wt% OBM slurry nanocomposite sample. Some peaks are noticeable between 400 to 700 cm^−1^, which can be attributed to metal oxide stretching vibration [32]. However, it is believed that adding a nanofiller that acts as a nucleating agent promotes a short-range ordering structure. It is apparent that this short-range ordering structure split the band at 718 cm^−1^, representing a monoclinic phase, and a weak peak emerged at 727 cm^−1^, representing the orthorhombic phase (highlighted by the red circle in Figure 6 and Figure 7) [33]. The presence of this new orthorhombic phase is attributed to the decreasing trend in the crystallinity of the LDPE/MMT and LDPE/OBM slurry nanocomposites, which is in agreement with the crystallinity data reported in our previous study [7,16,26]. It is interesting to notice in our previous report [26] that the percentage of crystallinity decreased predominantly in the nanocomposites with higher filler loading (5, 7.5 and 10 wt% filler contents), whereas, in this study, the orthorhombic phase only appeared for the nanocomposites with a similar polymer–filler composition. In addition, the tightly stacked MMT and OBM clay platelets restrict the polyethylene motion during crystallisation due to a reduced viscous flow between the lamellar clay tactoids. The constrained molecular motion in a confined lamellar region gives rise to the amorphous structures that were detected in the FTIR spectra at 1467 cm^−1^ [34]. It can be inferred, based on the spectra observation in Figure 7, that there is an absence of or an insignificant degree of chemical bonding between the LDPE polymer and OBM slurry. This characteristic indicates that only physisorption may be taking place between the OBM slurry platelets and LDPE polymer and that Van der Waals forces may occur between the filler and polymer. 

### 3.3. Tensile Properties of Neat LDPE, LDPE/MMT and LDPE/OBM Slurry Nanocomposites

**Figure 8:**
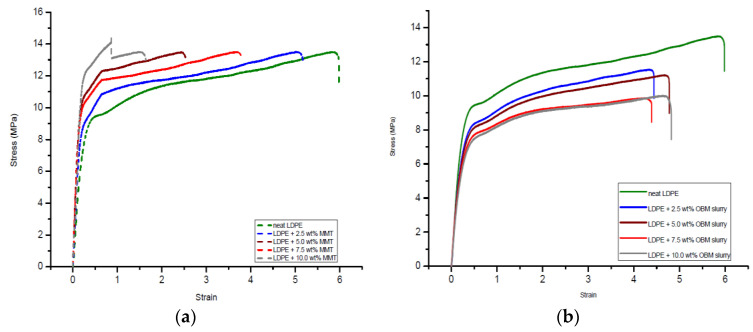
Stress–strain curves of (**a**) neat LDPE and LDPE/MMT and (**b**) neat LDPE and LDPE/OBM slurry nanocomposites.

The addition of the montmorillonite and OBM slurry fillers in the LDPE matrix resulted in changes in the mechanical properties of the LDPE/MMT and LDPE/OBM slurry nanocomposites. Observing the load-extension data from the tensile test of the LDPE/MMT and LDPE/OBM slurry nanocomposites (Figure 8), different mechanical properties were identified. 

**Figure 9:**
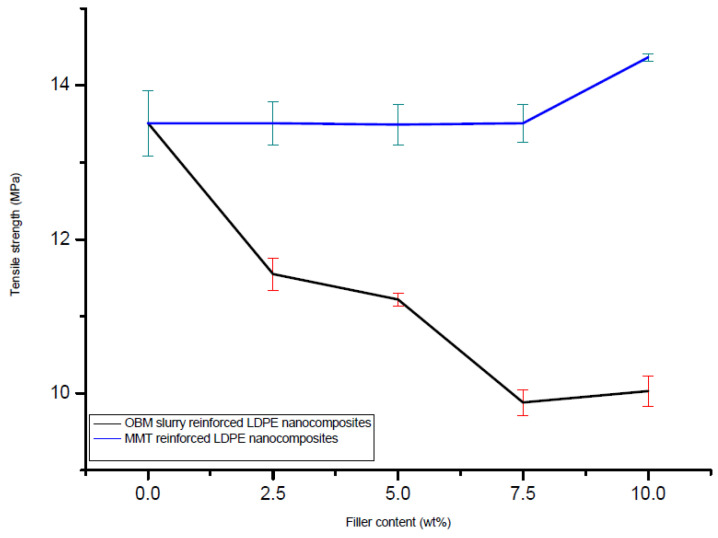
Comparison of tensile strength between LDPE/MMT and LDPE/OBM slurry nanocomposites considering tensile strength of neat LDPE as a baseline.

From the 7.5 to 10 wt% MMT loading in LDPE, there is a sharp increase in the tensile strength, which is noticeable in Figure 9, while only minor changes were observed in the tensile strength for 0 to 7.5 wt% MMT loading in LDPE. However, a progressive decrease in the tensile strength is highlighted in Figure 9 with increasing OBM slurry filler content from 0 to 10 wt%. This could be explained by the decohesion between the matrix and OBM slurry nanoparticles under stress. This decohesion generates a stress concentration through the void area between the matrix and nanoparticles, which accelerates the sample break. 

**Figure 10:**
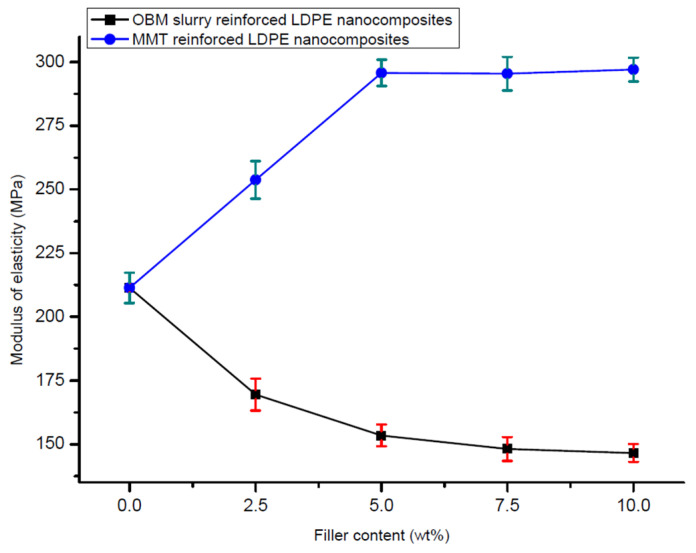
Comparison of modulus of elasticity in tension of neat LDPE, LDPE/MMT and LDPE/OBM slurry nanocomposites.

A sharp increase (~40%) in the modulus can be observed when the MMT loading increases from 0 to 5 wt%. Beyond 5 wt% in the MMT content, there are no significant changes noticeable in Young’s modulus (Figure 10). The opposite trend is evident for the LDPE/OBM slurry nanocomposites. A sharp decrease in the modulus (~30%) can be observed when the OBM slurry loading increases from 0 to 5 wt%. From 5 to 10 wt% of the OBM slurry loadings, there is no significant change in the elastic modulus. The addition of the MMT improved the stiffness of the LDPE matrix, whereas the stiffness decreased with the addition of the OBM slurry in the LDPE matrix, both at 5 wt% filler content. The load transfer mechanism between the clay platelets and polymer chain dictates the mechanical strength of the composite materials, and the adhesion strength between the clay platelets and polymer interface determines the degree of load transfer through the interface. However, the Young’s modulus is not affected much in most of the cases as small loads or displacements generally do not cause any delamination to determine the strength of the material. On the other hand, the strength and toughness properties of composite materials are directly related to the adhesion mechanism existing through the interface between organic and inorganic surfaces.

**Figure 11:**
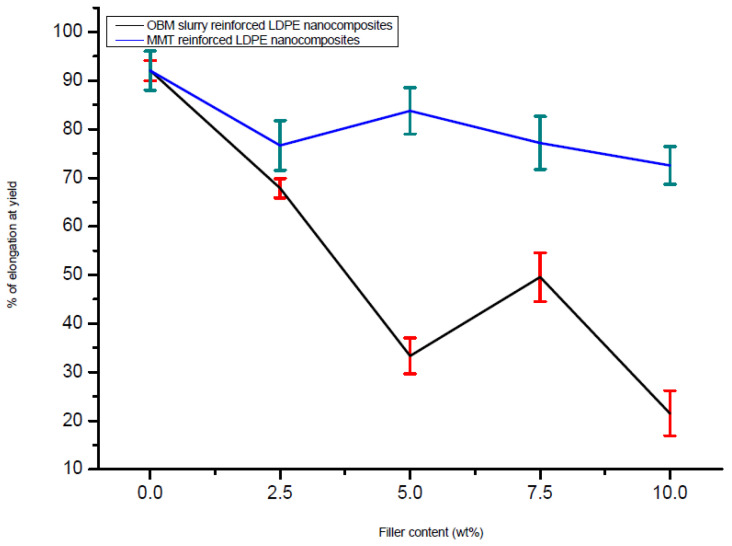
Comparison of elongation (%) at yield of neat LDPE, LDPE/MMT and LDPE/OBM slurry nanocomposites.

In Figure 9, the tensile strength decreases as the nanoclays loading increases in both the LDPE/MMT and LDPE/OBM slurry nanocomposites. In Figure 11, the opposite trend is noticeable in the LDPE/MMT nanocomposites, and the reason for this contradiction is that the properties of the composite material are not dependent only on the particle size and loading but interfacial adhesion also plays a vital role in controlling the properties in particulate composite material. The stress transfer between two phase components of composite material dictates the overall strength of the material. If the two phases are bonded together poorly, then the load transfer through the interface is inefficient and the strength of the material decreases as the loading of particulate material increases, which is highlighted by Fu et al. (2008) [35], Cho et al. (2006) [36] and Ahmed and Jones (1990) [37] in their studies. The theories and data observations in their studies also support the results shown in this study.

In Figure 10, it is noticeable that the Young’s modulus of the LDPE/MMT nanocomposites increases as the MMT loading increases, which reflects that the interfacial adhesion between MMT and LDPE is stronger than the interfacial adhesion between OBM slurry and LDPE. This phenomenon is true when a small load is applied to determine the stiffness of the materials. When the applied load exceeds the true elastic region, both the MMT and OBM slurry show similar trends in tensile strength, which is shown in Figure 9. However, the percentage of elongation at yield decreases with the increase in both the MMT and OBM slurry filler contents (Figure 11). 

There is a minor effect on the ductile property in the LDPE/MMT nanocomposites, which is presented in Figure 11, due to the addition of MMT loading in the LDPE matrix (~15% reduction). Nonetheless, the percentage of elongation at yield in the LDPE/OBM slurry nanocomposites decreases enormously (by up to ~80%) due to the addition of the OBM slurry in the LDPE matrix. It can be attributed to a reduction in the deformability of the interface between the nanoparticles and the matrix. From the morphology observation in Figure 4, the uniform distribution and dispersion of the OBM slurry clay fillers intensifies the crack propagation through the cross section of the samples and thus results in a reduced percentage of elongation in the LDPE/OBM slurry nanocomposites. The faster crack propagation in the LDPE/OBM slurry nanocomposites compared to that in the LDPE/MMT nanocomposites could be due to the close distances among the OBM slurry clay platelets, which influences the sample, leading to quicker failure even under lower forces applied on the samples. In addition, observing the cross section of the sample surfaces, it is noticeable that the fracture surfaces of the LDPE/OBM slurry nanocomposites are rough, whereas the surfaces of the LDPE/MMT nanocomposites are smoother than those of the LDPE/OBM slurry nanocomposites. The rough surfaces in the LDPE/OBM slurry nanocomposites indicate the poor interfacial adhesion, which also influences the percentage of elongation at yield point. 

### 3.4. Flexural Properties of Neat LDPE, LDPE/MMT and LDPE/OBM Slurry Nanocomposites

**Figure 12:**
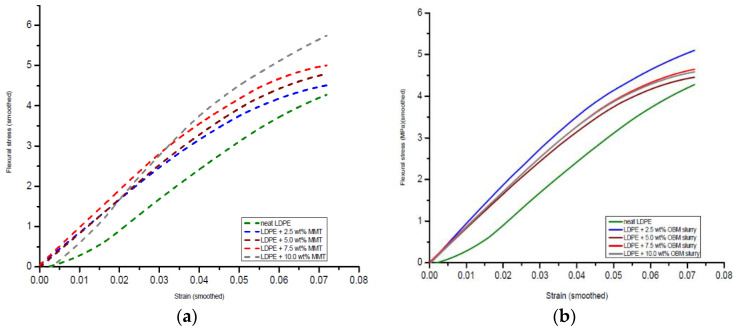
Flexural stress–strain curves from three-point bend test of (**a**) neat LDPE, LDPE/MMT and (**b**) LDPE/OBM slurry nanocomposites.

The flexural stress–strain curves of the neat LDPE, LDPE/MMT and LDPE/OBM slurry nanocomposites are shown in Figure 12.

It is observed that the gradient of the stress–strain curve in Figure 12a increases with the incremental loading of the MMT fillers in the LDPE matrix. The gradient of the stress–strain curve in Figure 12b was also higher for the LDPE/OBM slurry nanocomposites compared to that of the neat LDPE but was independent of the OBM slurry loadings. The constituents of the nanocomposite materials and the interface interactions between the matrix and reinforcement/filler are the key factors that dictate the flexural strength of fibre- or nanoparticle-reinforced nanocomposites [36]. Homogeneity is another important factor that needs to be considered when interpreting the flexural properties of materials. Since, in a flexural/bending test, the convex side of the sample is extended and the concave side is compressed, the degree of distribution of the clay nanoplatelets into the LDPE matrix also plays a key role in affecting the flexural properties. 

**Figure 13:**
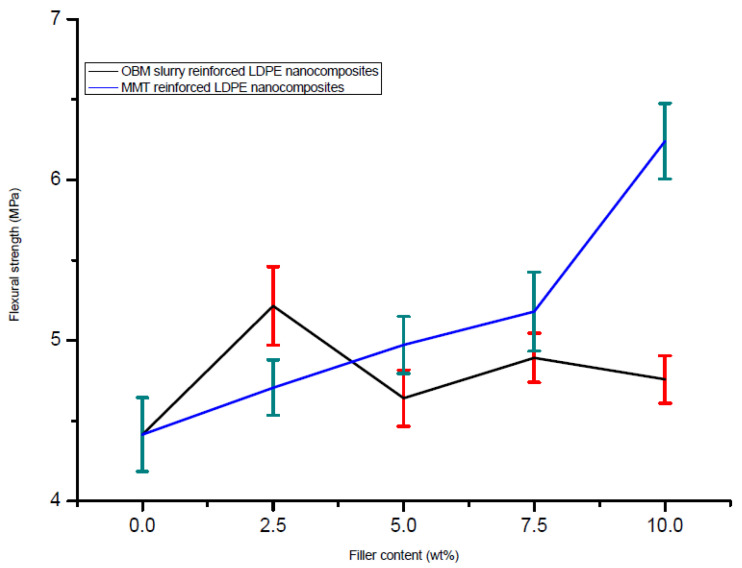
Comparison of flexural strength of LDPE/MMT and LDPE/OBM slurry nanocomposites.

The variation in the flexural strength of the LDPE/MMT and LDPE/OBM slurry nanocomposites reinforced with different weight (2.5, 5, 7.5 and 10) percentages of fillers is presented in Figure 13.

The neat LDPE shows the lowest flexural strength (4 MPa), which is considered as a reference point. There is a noticeable linear improvement in the flexural strength for the LDPE/MMT nanocomposites. However, the flexural strength does not show any trend for the LDPE/OBM slurry nanocomposites. The maximum flexural strength is evident for the LDPE/MMT nanocomposites with 10 wt% MMT content. However, the LDPE with 10 wt% OBM slurry nanocomposite shows the lowest flexural strength compared to the flexural strength of the other LDPE/OBM slurry nanocomposites.

**Figure 14:**
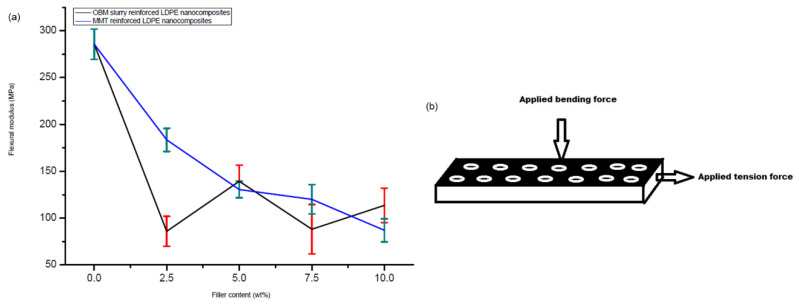
(**a**) Comparison of modulus of elasticity in bending of neat LDPE, LDPE/MMT and LDPE/OBM slurry nanocomposites; (**b**) a schematic illustration of applied force in tension and bending test and the distribution of MMT and OBM slurry nanoplatelets through the sample.

Furthermore, the flexural modulus decreased steadily in the LDPE/MMT nanocomposites from 0 to 10 wt% (Figure 14); however, it was not possible to correlate the effect of the OBM slurry loading to the flexural modulus of the LDPE/OBM slurry nanocomposites. Both the tensile and flexural modulus decreases with the incremental load of the OBM slurry in the LDPE matrix. Although the tensile modulus of the LDPE/MMT increases with the incremental loading of the MMT in the LDPE matrix, the flexural modulus of the LDPE/MMT nanocomposites decreases with the MMT loading from 0 to 10 wt%. 

The extent to which the reinforcing nanoplatelets influence the modulus of the materials depends directly on the thickness of the filler particles and the dispersion and distribution pattern of the nanoplatelets in the polymer matrix, and, thus, on the aspect ratio [38]. We have recently highlighted the nanomorphology, dispersion and distribution mechanism of the OBM nanoplatelets in the polymer matrix [7,16]. Further, we also reported an improvement in the thermal stability of the LDPE/OBMFs and PA6/OBMFs nanocomposites. Although the improvement in the thermal stability was achieved utilising both reclaimed clay (dry clay) from spent OBM waste and OBM clay in a raw suspended (OBM slurry) condition [7,16,26], the effect of this novel filler (OBM slurry in the raw condition) on the thermo-mechanical and mechanical properties was unknown. 

Based on the observations from these reported studies, it can be highlighted that the influence of the interfacial interaction between the polymer and clay governs the modulus property in the nanocomposites, which also agrees with the findings by Lee et al. (2009) [38]. The large interfacial area provides better stress transfer at the interface between the polymer and clay platelets. In Figure 14b, a schematic diagram illustrates the dispersion of the clay platelets into the polymer matrix. As the diameter of the white circle (depicting clay platelets) in Figure 14b is higher than its thickness, the interfacial area between the polymer and clay platelets is higher in the longitudinal direction. Hence, a higher tension load is needed to overcome the frictional force generated by the interfacial area between the polymer and clay platelets. On the other hand, in the three-point bending test, bending/flexural force is applied through the width of the sample and at the middle of the beam. As the thickness of the platelets is lower than its diameter, the frictional force at the interfacial area between the clay and polymer is lower than the frictional force generated in the longitudinal direction. This could account for the decreasing effect in the flexural modulus for both the LDPE/MMT and LDPE/OBM slurry nanocomposites.

Moreover, ATR-FTIR supports the formation of new chemical bonds due to the MMT reinforcement in the LDPE matrix, which plays a significant role in influencing the different mechanical properties studied in the LDPE/MMT nanocomposites. Even though the degree of dispersion and distribution of the OBM slurry in the LDPE matrix is superior compared to that of MMT, the lack of chemical bonding between the OBM slurry and LDPE polymer appears to impact the mechanical properties severely. The outcome of the cohesive law study based on the Van der Waals force at the atomic level by Tan et al. [39] and Frankland et al. [40] also supports the different mechanical results presented in this study. They reported in their study that carbon nanotubes did not form chemical bonds well, so the interfacial interaction mainly depends on the weak Van der Waals forces between carbon atoms and -CH_2_- units of polyethylene. Tan et al. [39] further presented how the effect of the nanoscale interfacial interactions between the filler and polymer influences the macroscopic behaviour of nanocomposites. They also highlighted how the cohesive stress increases rapidly at a lower opening displacement, which gradually decreases as the opening (completely debonded nanotubes) progresses further. The outcome of their study highlighted the generation of voids in the matrix due to the debonded nanotubes weakening the mechanical performance of the composite materials, which is also true for the results found in this study. 

### 3.5. Thermo-Mechanical Characterisation of Neat LDPE, LDPE/MMT and LDPE/OBM Slurry Nanocomposites

**Figure 15:**
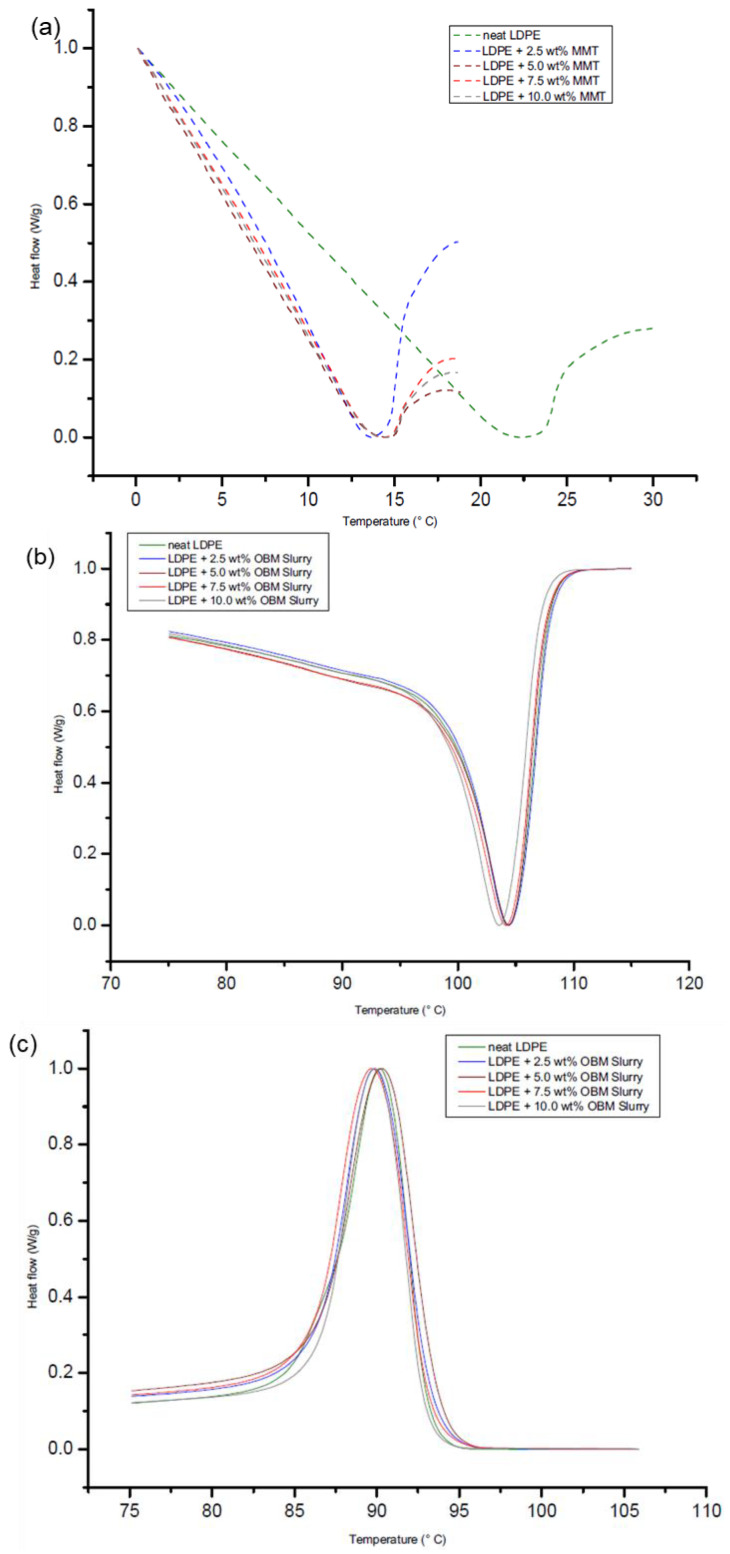
DSC thermograms of LDPE and LDPE/MMT nanocomposites at (**a**) glass transition temperature (Tg); and LDPE and LDPE/OBM slurry nanocomposites at (**b**) melting temperature (Tm) and (**c**) crystallisation temperature (Tc).

Detailed thermal properties of the LDPE, LDPE/MMT and LDPE/OBM slurry nanocomposites were reported in our previous work [26]. To identify the influence of the OBM slurry on the thermal degradation behaviour of the LDPE/OBM slurry nanocomposites, non-isothermal DSC studies were conducted. The investigation regarding the thermal degradation behaviour of the LDPE/MMT nanocomposites is considered as a benchmark standard. Analysing the DSC thermograms in Figure 15a, it can be highlighted that there are not any significant changes in the glass transition temperature (Tg) of the LDPE/MMT nanocomposite materials, but this Tg is lower than the Tg of neat LDPE. However, there are no significant changes among the Tg of the neat LDPE and LDPE/OBM slurry nanocomposites, which is shown in Figure 15. The same trend is noticeable in comparing the thermograms between the LDPE/MMT and LDPE/OBM slurry nanocomposites. The melting point remains almost the same for the neat LDPE and LDPE/OBM slurry nanocomposites, whereas the addition of the MMT filler lowered the melting point of the LDPE/MMT nanocomposites. 

The onset degradation temperatures of the neat LDPE, LDPE/MMT and LDPE/OBM slurry nanocomposite materials are presented at weight % losses of 5% and 10%. In each case, the onset degradation temperature of the nanocomposites is less than that of the neat LDPE. There are no significant changes in the D1/2 time (the time needed to reach 50% degradation), which indicates the filler content may not have any influence on the degradation time, and the increase in the filler contents in the nanocomposites may intensify the heat flow, which is demonstrated by the elevated temperature in the D1/2 time. It is also noticeable, for both nanocomposites, that the residue after 1000 °C increases with the incremental wt% of the fillers in the nanocomposites. It was observed that the LDPE/OBM slurry nanocomposites with higher percentage filler contents (7.5 and 10.0 wt%) appeared to act as thermally conductive materials. The heat capacity values of the nanocomposites decreased by about 33% in LDPE with 7.5 wt% MMT and about 17% in LDPE with 10.0 wt% OBM slurry. A significant difference in the residue amount (in %) left after the TGA in the two nanocomposites indicates that the OBM slurry has a significant influence in terms of decomposing the LDPE matrix.

**Figure 16:**
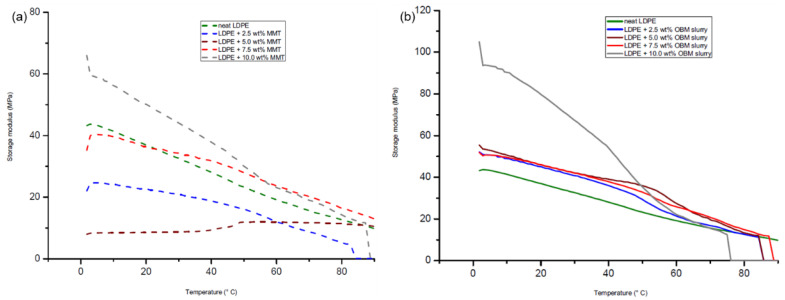
Influence of applied shear stress on the storage modulus of (**a**) neat LDPE and LDPE/MMT and (**b**) neat LDPE and LDPE/OBM slurry nanocomposites.

The thermo-mechanical properties of the neat LDPE, LDPE/MMT and LDPE/OBM slurry nanocomposites were studied using an oscillating shear rheometer to investigate the accumulated and percolated network of the fillers, which may influence the modulus of rigidity, electrical and thermal conductivity of the polymer nanocomposite materials. This approach is very effective at identifying the filler dispersion, structural behaviour of the materials and interaction of the filler in the polymer matrix. Figure 16 shows the dynamic temperature sweep analysis conducted to investigate the effect of the fillers on the storage modulus for the neat LDPE, LDPE/MMT and LDPE/OBM slurry nanocomposites.

The storage modulus (*E*′) highlights the load-bearing capacity of the neat LDPE, LDPE/MMT and LDPE/OBM slurry nanocomposites. It is noticeable from Figure 16a that the storage modulus of LDPE with 2.5 and 5 wt% MMT nanocomposites is lower than the storage modulus of the neat LDPE. However, the storage modulus of LDPE with 7.5 and 10 wt% MMT nanocomposites is higher than the storage modulus of the neat LDPE. LDPE with 10 wt% MMT nanocomposites demonstrates the highest storage modulus up to 50 °C and between 50 and 90 °C.

**Figure 17:**
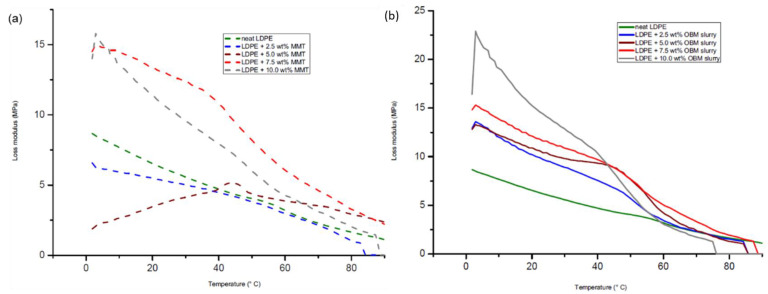
Influence of applied shear stress on the loss modulus of (**a**) neat LDPE and LDPE/MMT and (**b**) neat LDPE and LDPE/OBM slurry nanocomposites.

There is a minor difference in the storage modulus among the neat LDPE and LDPE/MMT nanocomposites, which is highlighted in Figure 16a. However, the storage modulus of the neat LDPE and LDPE/OBM slurry nanocomposites, presented in Figure 16b, shows the storage modulus of the LDPE/OBM slurry nanocomposites to be higher than the storage modulus of the neat LDPE up to the temperature of 50 °C. There is a minor storage modulus difference among the neat LDPE and LDPE/OBM slurry nanocomposites, which is highlighted between 50 and 90 °C in Figure 16a. From this observation, it can thus be concluded that the influence of the MMT and OBM slurry fillers on the storage modulus decreases with an increase in the temperature between 0 and 50 °C, and it is also noticeable that, from 50 to 90 °C, the storage modulus of the LDPE/MMT and LDPE/OBM slurry nanocomposites is filler-independent. The loss modulus curves of the neat LDPE, LDPE/MMT and LDPE/OBM slurry nanocomposites are presented in Figure 17, which shows that the relaxation peak of the neat LDPE shifted from 60 °C to lower temperatures due to the addition of fillers in the LDPE matrix.

In Figure 17a,b, there is a sharp decrease in the loss modulus between 0 and 50 °C for the neat LDPE, LDPE/MMT and LDPE/OBM slurry nanocomposites. However, there are very minor changes in the loss modulus that are noticeable between 60 and 90 °C. A similar trend as with the storage modulus for the LDPE/MMT and LDPE/OBM slurry nanocomposites is also evident for the loss modulus of the LDPE/MMT and LDPE/OBM slurry nanocomposites, which can be viewed as (i) the loss modulus of the LDPE/MMT nanocomposites: LDPE with 2.5 and 5 wt% MMT < neat LDPE < LDPE with 7.5 and 10 wt%; and (ii) the loss modulus of the LDPE/OBM slurry nanocomposites: neat LDPE < LDPE/OBM slurry nanocomposites. It is proposed that the Tg should be determined from the position of the tan δ peak and not the loss modulus peak.

**Figure 18:**
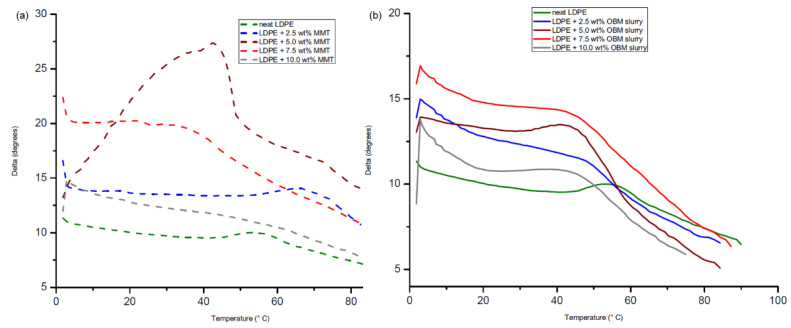
Influence of applied shear stress on damping property of (**a**) neat LDPE and LDPE/MMT and (**b**) neat LDPE and LDPE/OBM slurry nanocomposites.

To identify the damping properties of the materials, the ratio of loss modulus to storage modulus is calculated and presented in Figure 18a,b. 

The δ peaks in Figure 18a,b both showed an increased magnitude of δ compared to that of neat LDPE. This graphical representation highlighted the balance between the elastic and viscous phases in the polymeric structures. The highest δ peak of the neat LDPE shifted to lower temperatures for both the LDPE/MMT and LDPE/OBM slurry nanocomposites.

## 4. Conclusions

The objective of this study was to evaluate if the surfactants present in the OBM slurry may improve the interfacial adhesion between the OBM slurry and the LDPE matrix and thus improve the thermo-mechanical properties of new materials. The tensile, flexural and thermo-mechanical properties were investigated. It was observed that the addition of this novel filler was ineffective in improving the mechanical properties, and the mechanical strength of these new materials decreases significantly when compared to neat LDPE and LDPE/MMT nanocomposites as a benchmark standard. The morphology was studied in detail in previous research, and it demonstrated the improved dispersion capability of this filler after heat treatment compared to those of MMT [16], which also indicates a similar distribution and dispersion mechanism when the filler was used without any pre-treatment in this study. Furthermore, the tensile and flexural test data indicated that MMT intensifies the anisotropic properties in LDPE/MMT nanocomposites. The OBM slurry develops homogeneous dispersion throughout the LDPE matrix and indicates the non-structural delamination of the clay platelets in the LDPE matrix, thus showing isotropic properties in LDPE/OBM slurry nanocomposites. This statement is further validated by investigating the DMA of the LDPE/MMT and LDPE/OBM slurry nanocomposites. Although the addition of OBM slurry improves the thermal stability of LDPE composites, which was reported in our previous study [26], the results presented in this study highlighted the severe deterioration of the mechanical strength of LDPE composite material. It can be concluded here that the dispersion and distribution mechanism of the OBM clay is favourable, but the poor interfacial adhesion hinders the load transfer mechanism between particulate material and LDPE polymer chains. In addition, the storage modulus and loss modulus of LDPE/OBM slurry nanocomposites is higher than that of neat LDPE, which supports the observation regarding the non-structural delamination of the OBM slurry clay platelets in the LDPE matrix. The findings from this study encourage and can lead to the utilisation of this filler to turn these nanocomposites into ternary blending systems to improve different features of the materials, including the thermal, barrier and flame retardancy properties.

## Data Availability

Data available upon request.
